# Suppression of HIV in the first 12 months of antiretroviral therapy: a comparative analysis of dolutegravir- and efavirenz-based regimens

**DOI:** 10.31744/einstein_journal/2023AO0156

**Published:** 2023-05-23

**Authors:** Gabriella Jomara da Silva, Cássia Cristina Pinto Mendicino, Cristiane Aparecida Menezes de Pádua, Unaí Tupinambás

**Affiliations:** 1 Faculdade de Medicina Universidade Federal de Minas Gerais Belo Horizonte MG Brazil Faculdade de Medicina, Universidade Federal de Minas Gerais, Belo Horizonte, MG, Brazil.; 2 Faculdade de Farmáci Universidade Federal de Minas Gerais Belo Horizonte MG Brazil Faculdade de Farmácia, Universidade Federal de Minas Gerais, Belo Horizonte, MG, Brazil.

**Keywords:** HIV-1, Viral load, Sustained virologic response, Effectiveness, Dolutegravir, Efavirenz, Drug therapy, combination, Antiretroviral therapy, highly active, Anti-HIV agents

## Abstract

**Objective:**

To compare viral suppression in treatment-naïve adults starting antiretroviral therapy with dolutegravir (50mg)- and efavirenz (600mg)-based regimens.

**Methods:**

We analyzed secondary data from Brazilian health information systems of people living with human immunodeficiency virus who started antiretroviral therapy between 2015 and 2017 in Minas Gerais, Brazil. The outcome was viral suppression, defined as the achievement of the first viral load <50 copies/mL within 12 months after initiating antiretroviral therapy. This outcome was also compared with viral load <1,000 copies/mL and analyzed in two scenarios: intention-to-treat *versus* per-protocol. Time to viral suppression and adjusted odds ratio accompanied by 95% confidence intervals were estimated.

**Results:**

Of the 2,599 participants enrolled, 77.5% were men, and the median age was 34 years. In the intention-to-treat analysis, viral suppression was 58.1% for efavirenz and 76.7% for dolutegravir. People living with HIV on dolutegravir-based regimen were more likely to achieve viral suppression (aOR: 2.44; 95%CI: 2.01-2.95) and had a shorter median time to viral suppression (p<0.0001). Antiretroviral therapy initiation within <120 days, baseline CD4⁺T-cells ≥200 cells/mm^3^, and viral load <100,000 copies/mL had higher odds of viral suppression. According to the per-protocol analysis, viral suppression ≥90% was observed by considering viral load <1,000 copies/mL.

**Conclusion:**

Our study demonstrated that viral suppression improved after introducing dolutegravir, although the proportion of patients with viral load <50 copies/mL was lower than expected. Improved access to routine viral load examinations and continuous surveillance of the effectiveness of antiretroviral therapy should be considered.

**Figure:**
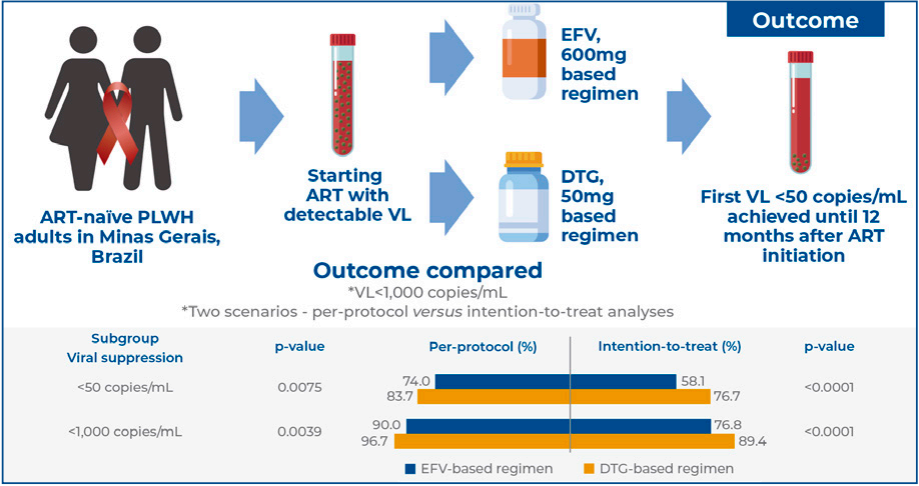

## INTRODUCTION

Antiretroviral therapy (ART) is crucial for increasing the survival of people living with the human immunodeficiency virus (PLWH) and preventing viral transmission. Viral suppression is one of the goals for eradicating the acquired immunodeficiency syndrome (AIDS) epidemic by 2030.^( 1 - 3 )^ Few countries reached the “90-90-90” targets by 2020;^( 4 )^ the constraints of the COVID-19 pandemic also negatively impacted treatment outcomes, suggesting the necessity for greater efforts in providing human immunodeficiency virus (HIV) care services.^( 5 , 6 )^Therefore, accessible healthcare infrastructure in low- and middle-income countries is needed; for instance, access to routine viral load (VL) testing can be expanded to efficiently monitor ART effectiveness.^( 7 - 10 )^

An undetectable VL is the main objective of HIV treatment. The World Health Organization (WHO) has been monitoring PLWH with VL <1,000 copies/mL to control the risk of transmission, disease progression, and therapeutic failure.^( 11 )^ In Brazil, viral suppression is monitored by the Department of Chronic Conditions and Sexually Transmitted Infections, by considering the threshold VL as <50 copies/mL, which is frequently considered the threshold value in clinical research.^( 12 - 16 )^

Few studies have assessed the proportion of individuals who have achieved viral suppression using first-line ART in Brazil,^( 13 , 17 - 19 )^ despite changes in the national HIV guidelines in 2017 that recommend the use of antiretroviral regimens with dolutegravir (DTG) as the first-line treatment and the use of efavirenz-based regimens (EFV) as the alternative first-line ART. Therefore, it is important to collect ample data at the regional level to identify obstacles in the achievement of sustainably undetectable VL.

## OBJECTIVE

To compare the viral suppression rates achieved with dolutegravir (50mg)- and efavirenz (600mg)-based regimens in the first 12 months of treatment and to evaluate the associated factors and time to viral suppression.

## METHODS

### Study population and variable definition

We conducted a historical cohort study of ART-naïve PLWH who started treatment with DTG- or EFV-based regimens between January 2015 and December 2017 in Minas Gerais, Brazil. The regimen containing DTG (50mg) was administered as two pills (a fixed-dose combination of tenofovir disoproxil fumarate (300mg) and lamivudine (300mg) plus other DTG pill), and the regimen containing EFV (600mg) was administered as one pill (a fixed-dose combination of tenofovir disoproxil fumarate (300mg), lamivudine (300mg), and EFV). Data were obtained from two administrative databases of the Unified Health System for HIV/AIDS of the Ministry of Health: the Medication Logistics Control System (SICLOM), which records the dispensation of ART, and the Laboratory Tests Control System (SISCEL), which records laboratory results of VL and CD4^+^ T-cell counts.^( 10 )^

The outcome was viral suppression, defined as the achievement of the first VL <50 copies/mL measured within 12 months after ART initiation. The proportion of individuals who achieved viral suppression was compared with an international parameter (VL <1,000 copies/mL).^( 11 )^Individuals with records of VLs measured at least once within 12 months after ART initiation and baseline VLs measured within 180 days before ART initiation were included in this analysis. Individuals who did not undergo VL examinations, had undetectable baseline VL (<50 copies/mL), or were aged <18 years were excluded. Explanatory variables included demographic, laboratory, and therapeutic variables, such as age, sex, place of residence, baseline VL, baseline CD4^+^ T-cell count, and time to ART initiation (defined as the time from the first CD4^+^T-cell examination until ART initiation). The patient informed consent was not essential for inclusion in this study, since an code number was used for patient identification and the data confidentiality was ensured at all stages of the project. The *Universidade Federal de Minas Gerais* (UFMG) Research Ethics Committee approved this study on January 19, 2017 (CAAE: 62710316.8.0000.5149; # 1.901.612).

### Data analysis

We conducted a descriptive analysis of the study population. Absolute/relative frequencies and median/interquartile ranges (IQRs) were estimated. The variables were categorized using the median or other criteria based on their clinical relevance. We compared demographic and clinical characteristics at baseline by using the χ^2^ and Mann-Whitney tests.

Time to viral suppression was defined as the time (in days) that elapsed from ART initiation until viral suppression was achieved, according to the first record of suppressed VL. For individuals who had not achieved viral suppression within 365 days, we considered the interval between the date of the first ART administration and the date when the last VL was >50 copies/mL or when ART was switched (censored data). Antiretroviral therapy switches were defined as alterations to any antiretroviral drug in the regimen or dose adjustments. Kaplan-Meier survival curves were generated to assess the time to viral suppression. The log-rank test was used to compare survival functions with a significance level of 0.05.

The analysis considered intention-to-treat (ITT) and per-protocol concepts to compare “real-world” and “controlled” scenarios of drug utilization, respectively.^( 13 )^ Intention-to-treat analysis included all the individuals who initiated first-line treatment regardless of whether they had switched or discontinued ART. The per-protocol analysis included individuals who were adherent to ART administered as first-line treatment and did not switch their ART. We selected possibly adherent individuals who had received ARTs 12 times within one year, since during the study period the Medication Dispensing Units commonly dispensed ART monthly. The variables were stratified into subgroups to assess the statistical differences in the occurrence of viral suppression according to the ART administered.

Univariate and multivariate analyses were performed using logistic regression to determine the association between viral suppression and putative risk factors. Variables with p≤0.20 in the univariate analyses were selected for inclusion in the final model. Backward stepwise regression was used to obtain the final model (variables with p<0.05) adjusted for age and sex to control for demographic characteristics, expressed as adjusted odds ratio (aOR) accompanied by 95% confidence intervals (95%CI). Model goodness-of-fit was assessed using the Hosmer-Lemeshow test. Analyses were conducted using the SAS software version 9.4 (SAS Institute Inc., Cary, NC, USA).

## RESULTS

### Population characteristics

**Figure 1:**
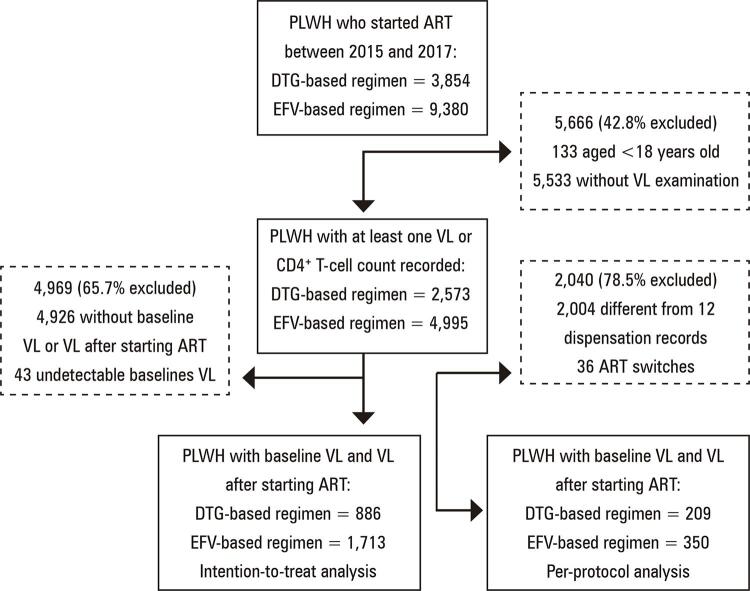
Flow diagram illustrating the patients included in the study according to data from the health databases ART: antiretroviral therapy; DTG: dolutegravir; EFV: efavirenz; PLWH: people living with human immunodeficiency virus; VL: viral load.

Between January 2015 and December 2017, 13,234 PLWH in Minas Gerais started first-line ART. We excluded 5,666 (42.8%) individuals who did not undergo VL examinations or were aged <18 years. Of the remaining 7,568 individuals, 65.7% were excluded because of the absence of VL records (n=4,926) or presence of undetectable VLs before starting ART (n=43). A total of 2,599 individuals were included in the ITT analysis (1,713 following the EFV-based regimen and 886 following the DTG-based regimen). The per-protocol analysis accounted for 559 individuals; 350 and 209 individuals were treated with EFV- and DTG-based ART, respectively ( Figure 1 ).

Most study participants were men (77.5%), and 57.8% of the participants lived in the countryside or neighboring cities. The median age was 34 years (IQR: 27-43), median baseline CD4⁺ T-cell count was 322 cells/mm^3^ (IQR: 146-507), and median baseline VL was 4.7 log_10_copies/mL (IQR: 4.2-5.3). The median time to ART initiation was 34 days (IQR: 20-71) and 17.7% of the included study participants took ≥120 days after the first CD4^+^ T-cell examination to start ART. Approximately 34% (n=886) and 66% (n=1,713) of the patients started first-line treatment with DTG-and EFV-based regimens, respectively ( Table 1 ). The median ART dispensation rate recorded during follow-up was higher in participants following the DTG-based regimen (12, IQR: 10-13) than in those following the EFV-based regimen (11, IQR: 9-13; p<0.0001). The median baseline CD4 **⁺** T-cell count and VL were 281 cells/mm^3^ (IQR: 121-461) and 4.9 log_10_copies/mL (IQR: 4.4-5.5), respectively, in PLWH following the DTG-based regimen and 343 cells/mm^3^ (IQR: 164-530) and 4.6 log_10_copies/mL (IQR: 4.1-5.2), respectively, in PLWH following the EFV-based regimen (p<0.0001).

**Table 1:** Baseline characteristics of adult people living with HIV and receiving initial antiretroviral therapy in Minas Gerais, Brazil, n=2,599

Characteristics	n (%)
Male sex, n (%)	2,013 (77.5)
Age (years), median (IQR)	34 (27-43)
Residence, n (%)	
State capital or metropolitan region	1,097 (42.2)
Countryside and others*	1,502 (57.8)
Baseline VL (log_10_copies/mL), median (IQR)	4.7 (4.2-5.3)
Baseline CD4⁺ T-cell count (cells/mm^3^)^†^, median (IQR)	322 (146-507)
Time to ART initiation (days)^†^, median (IQR)	34 (20-71)
Number of PLWH who initiated ART in ≥120 days, n (%)	461 (17.7)
Number of PLWH receiving ART, n (%)	
DTG-based regimen	886 (34)
EFV-based regimen	1,713 (66)

* PLWH living in neighboring cities; ^†^ 79 missing.

ART: antiretroviral therapy; DTG: dolutegravir; EFV: efavirenz; IQR: interquartile range; PLWH: people living with human immunodeficiency virus; VL: viral load.

### Viral suppression

**Figure 2:**
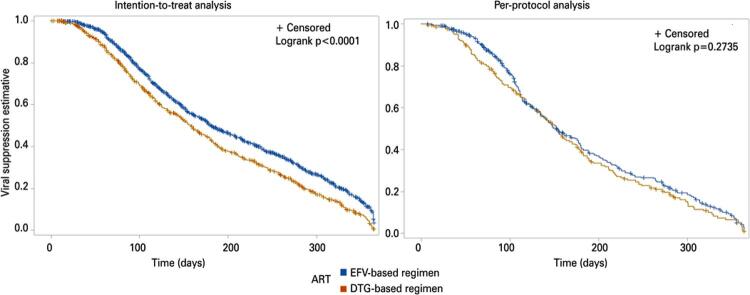
Kaplan-Meier analysis of time from antiretroviral therapy initiation to viral suppression. Viral suppression: first viral load<50 copies/mL achieved until 12 months after initiating antiretroviral therapy. The X-axis shows the time in days elapsed after starting antiretroviral therapy. The Y-axis shows proportions of individuals that achieved viral suppression ART: antiretroviral therapy; DTG: dolutegravir; EFV: efavirenz.

Figure 2 shows the Kaplan-Meier curves for the ITT and per-protocol analyses stratified by DTG and EFV-based regimens. According to ITT analysis, individuals starting first-line ART with the DTG-based regimen had a shorter median time to viral suppression than those with the EFV-based regimen (158 days [95%CI: 147-169] *versus* 181 days [95%CI: 174-195], p<0.0001). No significant differences were observed in the per-protocol analysis results (153 days for both regimens, p=0.2735).

**Figure 3:**
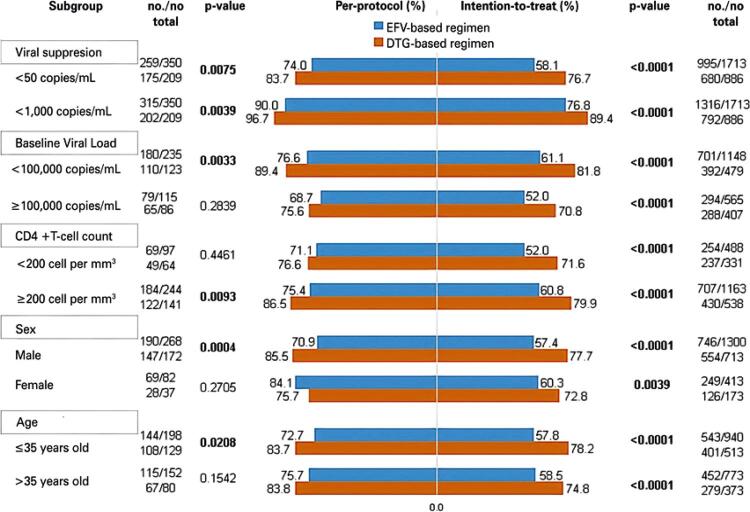
Subgroup analysis of the achievement of viral suppression within 12 months after initiating antiretroviral therapy

Among the 2,599 individuals included in ITT analysis, the percentage of PLWH on DTG- and EFV-based regimens with viral suppression (VL <50 copies/mL) was 76.7% (95%CI: 75.1-78.3) and 58.1% (95%CI: 56.8-59.4), respectively. Comparing PLWH with VL <1,000 copies/mL and those with VL <50 copies/mL (n=2,599), viral suppression was observed in 89.4% (12.7% increase) of the PLWH following the DTG-based regimen and in 76.8% (18.7% increase) of those following the EFV-based regimen. Viral suppression was observed in a higher percentage of PLWH following the DTG-based regimen than in those following the EFV-based regimen (p<0.05), even when stratified by age, sex, baseline CD4^+^ T-cell count, and baseline VL. Antiretroviral therapy switching in first-line treatment occurred in 5% and 9.2% of the PLWH following the DTG- and EFV-based regimens, respectively. According to the per-protocol analysis (n=559), viral suppression was observed in 83.7% and 74.0% of the PLWH following the DTG- and EFV-based regimens, respectively, with increases of 13.0% and 16.0%, respectively, when considering VL <1,000 copies/mL. No significant difference was observed between the ART regimen groups in terms of the percentage of PLWH with viral suppression when the CD4^+^ T-cell count <200 cells/mm^3^, sex was female, age was >35 years ( *i.e* ., older age), and baseline VL ≥100,000 copies/mL ( Figure 3 ).

Figure 3 shows a comparison of the number and percentage of participants with viral suppression (VL<50 copies/mL) in the first 12 months of ART and the first strata with VL <1,000 copies/mL. The variables were stratified into subgroups to assess the statistical differences in the occurrence of viral suppression according to the ART regimen followed. The left side of the figure corresponds to the per-protocol analysis while the right side corresponds to the intention-to-treat analysis.

### Univariate and multivariate analyses

According to both the univariate and multivariate analyses of the ITT analysis, the incidence of viral suppression was approximately 2.4-fold higher among individuals who initiated ART with a DTG-based regimen. The CD4^+^ T-cell count, baseline VL <100,000 copies/mL, and time to ART initiation were also maintained in the final model. In the per-protocol analysis, DTG-based regimen (aOR: 1.98; 95%CI: 1.27-3.09) and baseline VL <100,000 copies/mL (aOR: 1.85; 95%CI: 1.22-2.80) were independently associated with viral suppression ( Table 2 ).

**Table 2:** Results of the univariate and multivariate logistic regression analyses of predictors of viral suppression

Individual characteristics	Intention-to-treat analyses (n=2,599)				Per-protocol analyses (n=559)			
	
	Univariate analysis, OR (95%CI)	p value	Multivariate analysis, aOR (95%CI)^#^	p value	Univariate analysis, OR (95%CI)	p value	Multivariate analysis, aOR (95%CI)^†^	p value
Age								
>35 years	1				1			
≤35 years	1.05 (0.90-1.24)	0.5316	---	---	0.92 (0.62-1.39)	0.6988	---	---
Sex								
Male	1				1			
Female	0.98 (0.81-1.18)	0.7935	---	---	1.35 (0.81-2.25)	0.2543	---	---
Residence								
Countryside and others*	1		1		1			
State capital or metropolitan region	1.25 (1.06-1.47)	0.0080	1.18 (1.00-1.41)	0.0554	0.91 (0.61-1.35)	0.6259	---	---
Baseline viral load								
≥100,000 copies/mL	1		1		1		1	
<100,000 copies/mL	1.37 (1.16-1.62)	0.0002	1.48 (1.23-1.78)	<0.0001	1.69 (1.13-2.53)	0.0112	1.85 (1.22-2.80)	0.0037
Baseline CD4^+^T-cell count								
<200 cells/mm^3^	1		1		1			
≥200 cells/mm^3^	1.35 (1.13-1.60)	0.0007	1.37 (1.13-1.66)	0.0014	1.41 (0.92-2.17)	0.1143	---	---
ART								
EFV-based regimen	1		1		1		1	
DTG-based regimen	2.38 (1.98-2.86)	<0.0001	2.44 (2.01-2.95)	<0.0001	1.81 (1.17-2.80)	0.0080	1.98 (1.27-3.09)	0.0028
Time to ART initiation								
≥120 days	1		1		1			
<120 days	1.60 (1.30-1.96)	<0.0001	1.48 (1.19-1.85)	0.0005	1.54 (0.89-2.64)	0.1212	---	---

^#^ Hosmer-Lemeshow test: p=0.1240; ^†^ Hosmer-Lemeshow test: p=0.3295; P value bold type indicate that p<0.05; * individuals living in neighboring cities.

ART: antiretroviral therapy; 95%CI: 95% confidence interval; DTG: dolutegravir; EFV: efavirenz; OR: odds ratio; aOR: adjusted odds ratios.

## DISCUSSION

This study was performed using data from the public databases maintained by the HIV Surveillance System of National Monitoring, which enabled the assessment of the effectiveness of first-line ART among PLWH in Brazil. Minas Gerais occupied the fourth position in the Brazilian ranking of HIV infection incidence between 2007 and 2020.^( 20 )^ The profiles of the infected individuals in this study was similar to that of other studies conducted in Brazil, with a predominance of males and individuals aged between 30 and 49 years.^( 13 , 19 , 21 )^As expected, a higher proportion of men followed the DTG-based regimen compared to those that followed the EFV-based regimen, consistent with the Ministry of Health’s recommendations, which considered preliminary data that contraindicated DTG use in women with child-bearing potential.^( 22 )^

The introduction of fixed-dose combinations involving EFV plus lamivudine and tenofovir disoproxil fumarate demonstrated better results in terms of viral suppression within the first six months after initiating ART in the state capital, compared to multiple doses and drug formulations.^( 18 )^ Nevertheless, after the national HIV guidelines were amended to declare the DTG-based regimen as a first-line treatment, we observed higher odds of achieving viral suppression within 12 months after first-line with ART containing DTG. A historical cohort study in Brazil showed the superiority of DTG-containing ART by reporting that viral suppression (indicated by VL <50 copies/mL) was achieved in 90.5% of the cases that follow DTG-based regimens, even when controlling adherence.^( 13 )^This higher proportion of individuals with viral suppression compared to that in our study is due to methodological specificities and the high heterogeneity of the HIV-infected population across Brazil. Therefore, we highlighted the importance of analysis at the regional level.

We found that individuals who started treatment with the DTG-based regimen presented with higher baseline VL and lower baseline CD4^+^ T-cell counts. Despite these characteristics, viral suppression was achieved in a higher proportion of the PLWH following the DTG-based regimen compared with those following the EFV-based regimen. The superiority of DTG-containing ART over EFV-containing ART has also been verified in clinical trials;^( 15 , 16 , 23 , 24 )^ however, there was a lower difference 6% and 7% between the groups. Particularly in real-world scenarios, the improvement of ART adherence remains challenging,^( 8 , 21 , 25 )^ indicating the importance of monitoring adherence via readily accessible dispensation of existing drugs to achieve better treatment outcomes.

Higher switching rates were observed among individuals who started treatment with EFV-based regimens. Adverse effects and drug resistance leading to ART discontinuation have been reported more frequently with EFV-based regimens than with DTG-based regimens,^( 15 , 16 , 26 )^ which could explain the higher incidences of switching and lower annual dispensation rates observed with EFV-containing ART. Nervous system or psychiatric disorders are frequently reported among patients following an EFV (600mg)-based regimen.^( 15 , 26 , 27 )^ Brazil’s pharmacovigilance system has reported that the main adverse effects of DTG were nausea, diarrhea, and headache.^( 28 )^ Dolutegravir-based regimens have also been associated with nervous system disorders, such as insomnia;^( 16 , 29 - 31 )^ however, the rates of drug discontinuation are among the lowest in real-world scenarios.^( 29 )^The use of low-dose EFV (400mg) has demonstrated a reduction in ART discontinuation owing to adverse effects^( 32 , 33 )^ and this drug preparation has already been recommended by the World Health Organization as an alternative regimen.^( 34 )^Therefore, low-dose EFV could be considered as an alternative to first-line ART in low-and middle-income countries with a low prevalence of pretreatment non-nucleoside reverse transcriptase inhibitor (NNRTI) resistance, considering genotyped individuals without pretreatment resistance. Of note, EFV 400mg has not been made available in Brazil^( 35 )^ and the country is not among those with high pretreatment resistance to EFV.^( 36 )^ However, low-dose EFV would be indicated in genotyped individuals without pretreatment resistance.

According to the Joint United Nations Programme on HIV/AIDS (UNAIDS) mission to achieve the 90-90-90 target in Brazil, 85% of the PLWH knew their status, 77% could access treatment, and 94% exhibited viral suppression by 2018. In 2020, Brazil achieved percentages of 88-81-95, considering a VL of <1,000 copies/mL.^( 37 )^ In our study, achievement of viral suppression in ≥90% of the PLWH by 12 months was observed only when considering the same criterion (VL <1,000 copies/mL) and controlled adherence and/or changes in the first-line regimen. This finding can be reinforced by the data on diagnosed individuals who are not receiving adequate treatment. In addition, because even low but persistent viremia increases the risk of virological failure,^( 38 , 39 )^ this criterion may not indicate long-term therapeutic effectiveness.

A comparison of the per-protocol and ITT scenarios showed that higher proportions of individuals considered adherents exhibited viral suppression, and subgroup differences were detected to a lesser extent. Similarly, in a randomized, double-blind and phase III study, DTG-based regimens achieved viral suppression in a higher proportion of the study population than EFV-based regimens without significant differences between groups when baseline VL >100,000 copies/mL, baseline CD4⁺ T-cell count <200 cells/mm^3^, and the individuals were female and/or older.^( 16 )^ The NAMSAL study reported that a higher baseline VL (≥500,000 copies/mL) decreased the efficacy below 60% for both regimens, with a higher proportion of individuals receiving EFV-containing ART (400mg) achieving viral suppression compared with those receiving DTG-containing ART.^( 23 )^ Therefore, the predominance of certain baseline characteristics can influence the effectiveness of first-line ART. Another study observed that initiating ART within <120 days is a predictor of viral suppression.^( 40 )^ We observed that the individuals with baseline CD4⁺ T-cell count ≥200 cells/mm^3^ were more likely to achieve viral suppression, demonstrating that low immunological profile before starting ART may be an obstacle to viral suppression as shown in other studies.^( 8 , 13 , 25 , 41 , 42 )^ The association between baseline VL (<100,000 copies/mL) and viral suppression was observed in this study in both the ITT and per-protocol analyses. In addition, high baseline VL levels may be associated with viral blips and virological failures.^( 43 )^ Health services must recognize that these variables may affect therapeutic effectiveness and therefore prioritize the availability of antiretroviral drugs for individuals immediately after HIV diagnosis, in addition to routine monitoring and support for adherence.^( 25 )^

In the ITT analysis included in this study, individuals who initiated ART by following the DTG-based regimen achieved viral suppression earlier than those following the EFV-based regimen. However, no differences were observed in the per-protocol analysis, which does not reflect clinical practice. Another retrospective study observed that the median time to viral suppression was 60 days among individuals on DTG-containing ART or other integrase strand transfer inhibitors (INSTIs) compared to 137 days among those on EFV- or other NNRTI-based ART, when controlling adherence.^( 12 )^A randomized clinical trial observed that individuals achieved undetectable levels of VL in approximately 28 days with DTG-containing ART and in 84 days with EFV.^( 16 )^ The shorter time to achieve viral suppression is because VL is assessed more frequently in clinical trials than in routine clinical practice.^( 44 )^

PLWH who lived in the countryside or in other neighboring cities were significantly less likely to achieve viral suppression compared to those living in the state capital according to the univariate analysis. Others studies have shown that important differences in viral suppression can be observed depending on the place of residence or the size of the municipality.^( 45 , 46 )^ In addition, individuals living >200km away from the municipality of the pharmacy may be less likely to achieve viral suppression.^( 47 )^ Thus, it has been clearly demonstrated that the difficulty in accessing health services can influence the process of retention, adherence, and sustained viral suppression.

In this study, we observed that 93.4% (n=13,234) of the individuals started ART with EFV or DTG. Approximately 40% of the individuals did not have a VL record available for analysis. Minas Gerais has the greatest access to private healthcare (>20%) among all Brazilian states,^( 48 )^ thus justifying the absence of some of the data and preventing the estimation of more accurate viral suppression rates. VL monitoring is important for understanding the factors that prevent viral suppression, leading to virological failure^( 8 , 12 )^; however, it is not always performed in due course. In many low- and middle-income countries, PLWH have inadequate access to routine VL tests. Therefore, improving the patient’s access to VL monitoring and therapeutic education in clinical practice is of crucial importance.^( 8 )^

The ART regimens investigated in this study were selected in accordance with the current recommendations, which have been updated according to emerging evidence on their efficacy, effectiveness, and safety. Recent studies have reported that metabolic abnormalities, including hyperglycemia^( 49 - 51 )^ and weight gain,^( 23 )^ are associated with DTG. Among PLWH with tuberculosis, the use of twice-daily DTG rather than EFV is recommended when pretreatment genotyping within less than two weeks is not possible.^( 52 )^ In pregnant PLWH, the current data detailing the low incidence of DTG-induced neural tube defects have been reassuring so far.^( 34 )^Nevertheless, continued surveillance of the association of DTG-based regimens with neural tube defects and metabolic alterations is required. Additionally, DTG is more expensive than EFV;^( 53 )^ however, DTG-based regimens may be more cost-effective.^( 54 , 55 )^ In this study, we collected data from the period before the COVID-19 pandemic as the pandemic-imposed constraints on HIV care services may influence our results otherwise.

Nevertheless, this study has some limitations. Assuming higher rates of viral suppression with DTG-based regimens among individuals linked to private health centers, our results may have been underestimated. Viral load examination results of individuals followed in the private health network are often not filled in ART prescription forms, which prevents recording to SICLOM during the ART dispensing. In addition, the records of the VL examinations represent access to the examinations rather than the actual moment of viral suppression. Therefore, the time required for viral suppression should be cautiously interpreted. Despite these limitations, our study adds to the real-world evidence of the benefits of DTG-based regimens, thus providing guidance for future clinical decisions. Further larger and long-term studies that evaluate the routine clinical practice are required to assess the ART effectiveness given the constant update of national HIV guidelines, but it will mainly require improvements in access and recording VL examinations.

## CONCLUSION

In conclusion, viral suppression was more likely to be achieved or achieved earlier with dolutegravir-based regimens in a real-world scenario. Some baseline characteristics, such as early initiation of antiretroviral therapy, high CD4^+^T-cell count, and low viral load, were predictors of viral suppression, although first-line antiretroviral therapy with dolutegravir exhibited greater effectiveness, even with worse immunological and virological patient profiles, compared with efavirenz. Higher rates of viral suppression were attained with efavirenz-based regimens when adherence and antiretroviral therapy alterations were controlled (per-protocol analysis); however, these rates were lower than those attained with dolutegravir-based regimens. Nevertheless, the incidence of viral suppression (viral load<50 copies/mL) was lower than the global target, which reinforces the importance of evaluating treatment guidelines and their impact on clinical outcomes in real-world scenarios as well as improving public access to routine viral load examinations.
